# Postoperative bacteremia in periodontal flap surgery, with and without prophylactic antibiotic administration: A comparative study

**DOI:** 10.4103/0972-124X.65430

**Published:** 2010

**Authors:** Kanwarjit S. Asi, Amarjit S. Gill, Sunil Mahajan

**Affiliations:** *Department of Periodontics, Krishna Dental College, 95 Loni Road, Mohan Nagar, Ghaziabad, UP, India*; 1 *Director Principal, Surendra Dental College & Research Institute, H.H. Gardens, Sri Ganganagar, Rajasthan - 335 001, India*; 2 *Institute of Dental Sciences, Sehora, Jammu J&K, India*

**Keywords:** Postoperative bacteremia, periodontal flap surgery, prophylactic antibiotic administration

## Abstract

**Objectives:**

Many a times in clinical periodontology, the decision whether to prescribe prophylactic antibiotics or not, is perplexing.The present study was conducted to compare the bacteremias induced after periodontal flap surgeries with and without prophylactic antibiotics.

**Materials and Methods:**

The occurrence of postoperative bacteremia following periodontal flap surgery was studied in 30 patients. On these patients, 30 quadrant wise flap surgeries were carried out without any preoperative prophylactic antibiotics and 30 surgeries carried out after prophylactic administration of amoxycillin preoperatively. A blood sample was taken from each patient at the time of maximum surgical trauma and was cultured for micro-organisms and antibiotic sensitivity.

**Results:**

18 out of 60 blood samples were positive for micro-organisms. There was a significant reduction in post operative bacteremia after amoxycillin prophylaxis (x^2^ - 7.96 with *P*<0.01) as post operative bacteremia was found in 14 of the non medicated patients as compared to only 4 of the pre medicated patients. The micro-organisms encountered in the study are as follows:- 1) *Staphylococcus albus coagulase negative, 2) Klebsiella, 3) Psedomonas aerugenosa, 4) Streptococcus viridans, 5) Alpha hemolytic *streptococcus*, 6) *Neisseria catarrhalis**

**Conclusion:**

On the basis of the study, it is concluded that the incidence of postoperative bacteremia following periodontal flap surgery is not as high as previously reported. The clinical results show that Amoxicillin is highly effective in reducing postoperative bacteremia in periodontal flap surgery and thus in preventing the possible sequelae (Infective Endocarditis and other systemic maladies) in susceptible patients. However, cefotaxime and cephalexin may prove to be more effective in preventing the same.

## INTRODUCTION

Bacteremia has been defined as the presence of bacteria in blood. Bacteremia of dental origin has been attributed to periodontal infection, tooth extraction, periodontal scaling, gingivectomies, root canal therapy, gingival massage, use of oral irrigation devices, mastication, rocking of teeth and the administration of local anesthesia. Throughout the literature there are inconsistencies regarding the incidences of bacteremia attributed to any one cause[1].

Transitory bacteremia, induced by oral procedures, may result in bacterial endocarditis in patients with congenital cardiac defects or acquired heart valve abnormalities. An increasing number of patients have prosthetic cardiovascular appliances and are also vulnerable to serious complications from a transitory bacteremia[2].

Prophylactic use of antibiotics in surgery of the oral cavity is becoming standard procedure in order to minimize bacteremia and its deleterious effects. The unnecessary use of antibiotics results in the development of resistant strains of bacteria. On the other hand, there are instances when the prophylactic use of antibiotics is overlooked because of failure to establish its need. In order to solve thi s dilemma, the present study was done to compare the bacteremia induced by the periodontal surgery with and without administration of prophylactic antibiotics.

## MATERIALS AND METHODS

This investigation was carried out on 30 patients in the 35-45 years age group at the Department of Periodontology and Oral Medicine, Punjab Government Dental College and Hospital, Amritsar, Punjab.

The diagnosis of chronic generalized periodontitis and the need for periodontal flap surgery was determined after clinical and roentogenographic examination. All the patients were treated in the same manner. The patients were put on plaque control, which included brushing and rinsing the mouth with 0.02 % chlorhexidin mouth wash twice daily. Full mouth scaling was accomplished by hand instruments followed by polishing with rubber cup and polishing paste. Routine laboratory investigations comprising blood for hemoglobin, bleeding time, clotting time, total leukocyte count, differential leukocyte count and urine for complete examination were carried out. Four quadrant-wise, flap surgeries were performed in 30 patients each. Of these, one quadrant wise periodontal flap surgery from each patient was performed without any prophylactic antibiotic and was labeled series I while the other three quadrant-wise periodontal flap surgeries in each patient were carried out after prophylactic administration of 500 mg Amoxicillin orally, two hours prior to the surgery. Of the three, one quadrant-wise periodontal flap surgery was labeled series II and included in the study. The study was set up so that the patients participated twice, in this way they could serve as their own controls.

Modified Widman flap (Ramfjord and Nissle, 1974) was performed quadrant-wise in each of the participants.

**Figure 1:**
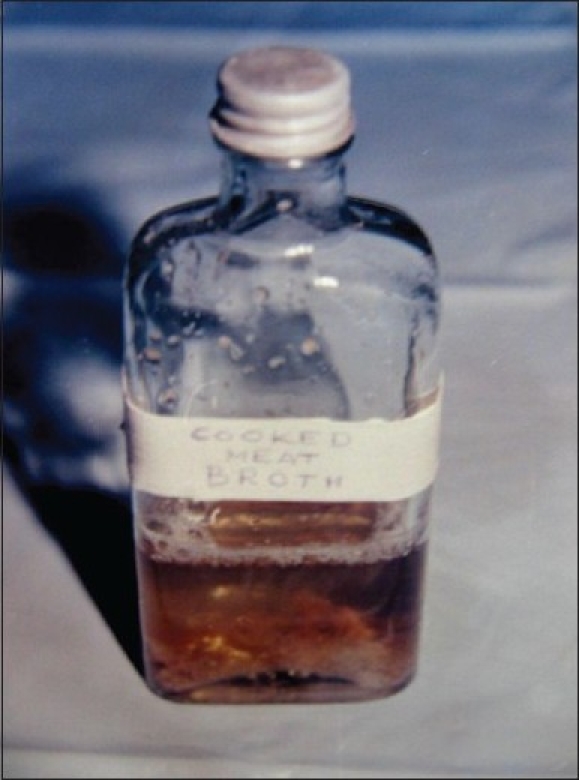
Brain-heart infusion broth with cooked meat particles

The blood was collected in the following manner during the flap surgery at the stage of degranulation, scaling and root planing, which was designed to be the point of maximum trauma. 10 (TEN) cc of blood was withdrawn from a vein in the cubital fossa. The blood was then transferred into already labeled bottle containing 50cc of Brain Heart Infusion broth with cooked meat particles [Figure 1], making a liberal use of the flame during the procedure. The bottles were incubated at 37°C for 18-24 hours.

The ‘Z’ test for proportions was applied to the results of the study. This follows the standard normal distribution. This calculated value of Z i.e. 3.07 was greater than the critical value 1.96 at 5% level of significance and even at 1% level of significance. This shows that the reduction of the bacteremia following periodontal flap surgery after prophylactic administration of 500mg of amoxicillin is highly significant. The significance of the data of the study was also tested by means of the chi square test, appropriate for a 2 × 2 table. The demonstration of x^2^ = 7.96 with *P*<0.01 indicates a highly significant reduction in the incidence of post operative bacteremia in the patients given amoxicillin prophylactically.

Of the 60 samples taken, 18 i.e. 30% were positive for micro-organisms. Of these 14, positive cultures were obtained in series I, a percentage of 46.6% and four positive blood cultures in series II, a percentage of 13.3%. A comparison of 14 positive cultures (46.6%) in series I to four positive cultures (13.3%) in series II shows a significant inhibition of bacteremia following premedication with amoxicillin.

**Figure 2:**
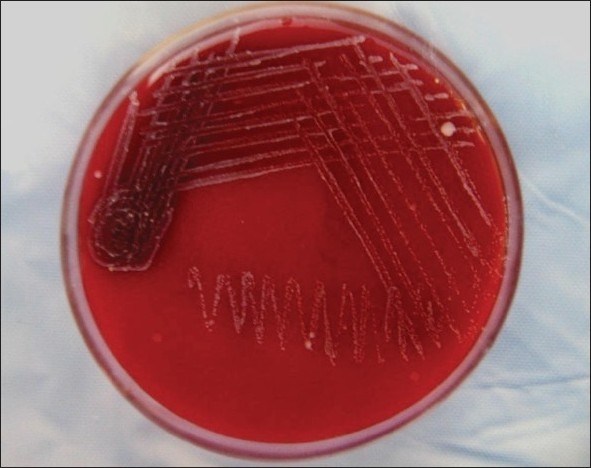
Culture plate showing growth of *Staphylococcus* albus coagulase negative

**Figure 3:**
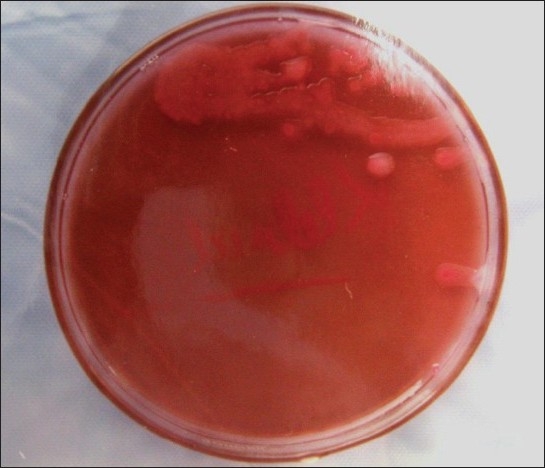
Culture plate showing growth of *Klebsiella*

**Figure 4:**
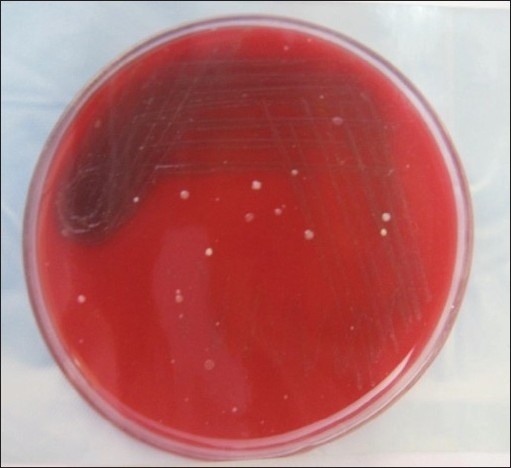
Culture plate showing growth of *Streptococcus* viridans

The following micro-organisms were encountered [Table 1]:

1. *Staphylococcus albus* coagulase negative [Figure 2]
2. *Klebsiella* [Figure 3]
3. Pseudomonas aerugenosa
4. *Sreptococcus viridans* [Figure 4]
5. Alpha hemolytic *streptococcus*
6. Neisseria catarrhalis

**Table 1:** Frequency of occurrence of micro-organisms in series I and II

Organisms	Frequency of occurrence		Total
	Series 1 (without preoperative prophylactic antibiotics)	Series 1 (with preoperative prophylactic antibiotics)	
*Staphylococcus albus*	6 (20)	0	6 (10)
coagulase negative *Klebsiella*	1 (3.3)	2 (6.6)	3 (5)
*Pseudomonas aerugenosa*	2 (6.6)	1 (3.3)	3 (5)
*Streptococcus viridans*	2 (6.6)	1 (3.3)	3 (5)
Alpha hemolytic *streptococcus*	2 (6.6)	0	1 (3.3)
*Neisseria catarrhalis*	1 (3.3)	0	1 (1.6)
**			
**			

Figures in parenthesis are in percentage

*Staphylococcus albus* was the most frequently isolated micro-organism occurring six times and *Neisseria catarrhalis*, the least isolated, occurring only once. Unlike previous reports, *streptococcus viridans* was not the most frequently isolated micro-organism.

**Figure 5:**
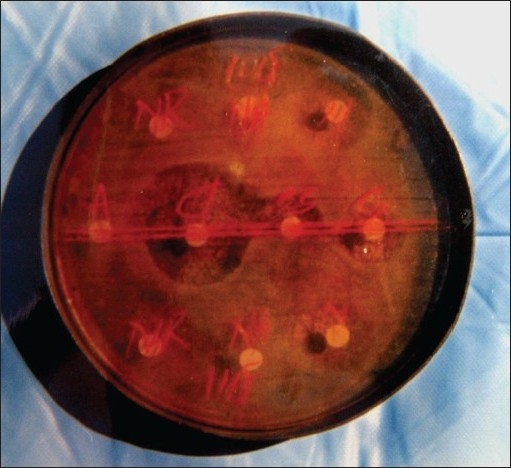
*In vitro* antibiotic sensitivity test

Prophylactic amoxicillin was effective against all the micro-organisms except *Klebsiella*, whose frequency, increased after premedication with amoxicillin. *In vitro* antibiotics sensitivity tests [Figure 5] show that cefotaxime and cephalexine were the most effective of the antibiotics tested, each of the isolated micro-organism being sensitive to the other antibiotics in decreasing order of their spectrum of activity are as follows; [Table 2]

**Table 2:** Incidence of bacteremia in series I and II

	Series 1 (Without preoperative prophylactic antibiotics)	Series 1 (With preoperative prophylactic antibiotics)	Total
Number of surgeries	30	30	60
Frequency of bacteremia	14	4	18
Percentage	46.66	13.33	30

Ciprofloxacin
Amoxicillin
Erythromycin
Gentamycin
Norfloxacin

## DISCUSSION

During periodontal surgery, the microbial challenge to the patient is enormous. The occurrence of post surgical bacteremia varies with amount of trauma inflicted. Hence the lengthy periodontal procedures, particularly involving surgical trauma, may be associated with a high percentage of transient bacteremia. The opinion regarding the occurrence of bacteremia following various non surgical and surgical manipulations of the oral tissues is varied.

Recognizing the possibility of bacteremic conditions existing in patients, prior to above mentioned procedures, the desirability of taking pre operative blood samples was considered. The literature review, however, suggested that this was unnecessary. Frequently investigators taking pre operative blood samples early in their work discontinued the procedure after a long series of negative result.[3]

In the present study, 30% of the 60 blood cultures were positive for micro-organism after a periodontal flap surgery. Of these 60 blood cultures, 30 of the non prophylactically medicated cases, bacteremia was encountered in 46.6% cases. When compared with previous studies these occurrences are fairly low. In 1960, Rogosa *et al*,[4] demonstrate that 88% of all blood cultures are positive after periodontal procedures. As regards to other surgical procedures in the oral cavity, Okell and Elliott[5] found that 60.9% of their cases showed bacteremia after extractions, carried out under general anesthesia.

Lazansky, Robinson and Rodofsky[6] have reported the lowest incidence of bacteremia is in the age group of 30-59 years. This could, in part, explain the low incidence of bacteremia in the present study as the age of the participants falls within the above mentioned age group.

Moreover, the preparation of the patients by scaling and institution of oral hygiene measures could have drastically reduced the number of micro-organisms in the oral cavity thus reducing the chances of post operative bacteremia.

In the present study, half of the surgeries were performed with antibiotic prophylaxis, further reducing the incidence of bacteremia. We used local anesthesia (2% lignocaine) with 1: 200000 adrenalin. As adrenalin is a potent vasoconstrictor, this could partially explain the low incidence of bacteremia in the present study.

The blood sample was taken at the time of maximum trauma, which usually occurred after 20 minutes of the initial incision. The incidence of bacteremia is highest in the 20-60 minute interval, according to Vargas *et al*.[7] However, others like Lazansky, Robinson and Rodofsky[6] have shown that no bacteremia was detected after 10 minutes from the time of starting of instrumentation. If the latter study is to be believed, this time factor may also be responsible for lower incidence of bacteremia in the present study.

‘Rocking of teeth’ as mentioned by Coffin and Thompson[8] is a major factor in the causation of bacteremia after exodontia, which was not the case in the present study, thus accounting for reduced incidence of bacteremia. About 46.6% bacteremia noticed in the study in the nonprophylactically medicated group - series I is, however, comparable to post extraction bacteremia reviewed by Khairat[9] in which he reported, Marseille– 42%, Cooley and Haberman– 39%, Hirsch *et al*,– 46% had positive blood cultures.

In the prophylactically medicated patients i.e. series II, the occurrence of bacteremia was 13.3%. The present study shows a highly significant reduction in the occurrence of positive blood cultures in patients given amoxycillin prophylactically. The value of χ^2^ = 7.96 with *P*<0.01 by chi square test, for present study is contradictory to the results obtained by Appleman, Suttar and Sims[10] who reported non significant reduction (*P*<0.8) in the incidence of positive blood culture after cephalexin premedication following periodontal surgery.

*Streptococcus viridans* has been documented to be the most frequently encountered micro organism responsible for post operative bacteremia by various authors.[5,7] However, in the present study *Staphylococcus albus* coagulase negative was the most frequently isolated micro-organism.

Okel and Elliot[5] considered *Staphylococcus albus* coagulase negative as contaminants. However, McEntegart and Porterfield[11] considered *Staphylococcus albus* coagulase negative as pathogenic micro-organisms. *Staphylococcus albus* may cause septicaemia in immuno suppressed patients, they often cause a bacteremic illness in patients treated with an indwelling venous catheter / instrumentation. It can also cause endocarditis in susceptible patients.[12]

De leo *et al*,[13] have reported an incidence of three positive cultures for *Staphylococcus albus* out of 28 blood cultures of pediatric patients undergoing prophylaxis, a percentage of 10 % approximately which is in conformity with our observation.

There was a total inhibition of *staphylococcus albus* by preoperative amoxycillin prophylactically as compared to six times in the non premedicated group.

It is of interest to know that *Klebsiella* was recovered twice in patients given Amoxicillin prophylactically as compared to once in the non premedicated group. This could be because;

- *In vivo* and *in vitro* sensitivity of various strains of *Klebsiella* may vary
- The dose of Amoxicillin may not have reached the required levels in the blood.
- The strain may be resistant to the antibiotics *in vivo*,
- Majority of the *Klebsiella* isolates are resistant to ampicillin and carbenicillin. It may also be resistant to amoxycillin because of same mode of action. Hospital strains of *Klebsiella* display multiple resistance[14]
- As Amoxicillin was effective against other micro-organisms and not against *Klebsiella* it could have disturbed the symbiotic relationship of the oral micro-organisms giving *Klebsiella* an opportunity for proliferation and thus entering the stream more often.

Khairat[9] reported one positive blood culture out of 155 samples for *Klebsiella* species whereas in our study it occurred three times in a total of 60 samples.

In the present study, pseudomonas was encountered as pathogenic micro organism in 5% of the blood samples. It occurred twice in non premedicated patients and was encountered once in patients administered amoxicillin prophylactically.

Gutverg and Haberman[14] isolated pseudomonas from the periodontal pockets of 5 out of 231 patients. In this study, *streptococcus viridans* was encountered in three of the 60 blood samples, occurring twice in non premedicated patients and once in patients given prophylactic amoxicillin. This finding is contradictory to the results of several authors namely Vargas *et al*, Bender *et al* and Khairat, who have reported a high incidence for *streptococcus viridans* after various surgical and nonsurgical manipulation of the oral tissues.

Alpha hemolytic *streptococcus* occurred only two times in the present study. It was never encountered in the premedicated group, but occurred twice in the non premedicated group, only. Alpha hemolytic *streptococcus* was isolated 21 times in 22 positive blood samples out of 221 operations (Extraction and Periodontal Scalings) by Lazansky, Robinson and Rodofsky.[6] So according to them Alpha hemolytic *streptococcus* was the most frequently found organism which is not the case in the study. *Neisseria catarrhalis* occurred only once in the present study, in the non medicated patients and never occurred in the patients given preoperative amoxicillin. Khairat[9] encountered this micro-organism only once in 100 blood samples. Vargas *et al*,[7] isolated *Neisseria catarrhalis* from 90 out of 114 gingival sulci, but never from the blood samples. Its high incidence in gingival sulci could possibly explain the presence of this micro-organism in the blood.

## CONCLUSIONS

On the basis of the study, it is concluded that the incidence of postoperative bacteremia following periodontal flap surgery is not as high as previously reported. The causes for this finding could be the age group of the patients, pre operative preparation of the patients, antibiotics prophylaxis and the vasoconstriction at the operative site owing to the adrenalin content of the local anesthetic used. The clinical results show that Amoxicillin is highly effective in reducing postoperative bacteremia in periodontal flap surgery and thus in preventing the possible sequelae (Infective Endocarditis and other systemic maladies) in susceptible patients. It is concluded that pre operative prophylactic administration of antibiotic is a pre requisite to prevent the postoperative bacteremia and its possible sequelae following periodontal surgery. Amoxicillin is highly effective in preventing such post operative bacteremia. However, cefotaxime and cephalexin may prove to be more effective in preventing the same.
